# Brown Adipose Tissue Response Dynamics: *In Vivo* Insights with the Voltage Sensor ^18^F-Fluorobenzyl Triphenyl Phosphonium

**DOI:** 10.1371/journal.pone.0129627

**Published:** 2015-06-08

**Authors:** Igal Madar, Elinor Naor, Daniel Holt, Hayden Ravert, Robert Dannals, Richard Wahl

**Affiliations:** Division of Nuclear Medicine, The Russell H. Morgan Department of Radiology, The Johns Hopkins Medical Institutions, Baltimore, MD, United States of America; Institute of Zoology, CHINA

## Abstract

Brown adipose tissue (BAT) thermogenesis is an emerging target for prevention and treatment of obesity. Mitochondria are the heat generators of BAT. Yet, there is no noninvasive means to image the temporal dynamics of the mitochondrial activity in BAT *in vivo*. Here, we report a technology for quantitative monitoring of principal kinetic components of BAT adaptive thermogenesis in the living animal, using the PET imaging voltage sensor ^18^F-fluorobenzyltriphenylphosphonium (^18^F-FBnTP). ^18^F-FBnTP targets the mitochondrial membrane potential (ΔΨm)—the voltage analog of heat produced by mitochondria. Dynamic ^18^F-FBnTP PET imaging of rat’s BAT was acquired just before and during localized skin cooling or systemic pharmacologic stimulation, with and without administration of propranolol. At ambient temperature, ^18^F-FBnTP demonstrated rapid uptake and prolonged steady-state retention in BAT. Conversely, cold-induced mitochondrial uncoupling resulted in an immediate washout of ^18^F-FBnTP from BAT, which was blocked by propranolol. Specific variables of BAT evoked activity were identified and quantified, including response latency, magnitude and kinetics. Cold stimulation resulted in partial washout of ^18^F-FBnTP (39.1%±14.4% of basal activity). The bulk of ^18^F-FBnTP washout response occurred within the first minutes of the cold stimulation, while colonic temperature remained nearly intact. Drop of colonic temperature to shivering zone did not have an additive effect. The ß3-adrenergic agonist CL-316,243 elicited ^18^F-FBnTP washout from BAT of kinetics similar to those caused by cold stimulation. Thus, monitoring ΔΨm *in vivo* using ^18^F-FBnTP PET provides insights into the kinetic physiology of BAT. ^18^F-FBnTP PET depicts BAT as a highly sensitive and rapidly responsive organ, emitting heat in short burst during the first minutes of stimulation, and preceding change in core temperature. ^18^F-FBnTP PET provides a novel set of quantitative metrics highly important for identifying novel therapeutic targets at the mitochondrial level, for developing means to maximize BAT mass and activity, and assessing intervention efficacy.

## Introduction

The recent discovery of metabolically active brown adipose tissue (BAT) depots in human adults [1–5] has opened new avenues for the search of therapeutic approaches to the prevention and treatment of obesity and comorbidities (e.g., diabetes, heart disease). BAT is unique in its capacity to dissipate a huge amount of caloric energy into heat, 300 times more than an equivalent volume of any other tissue [6]. BAT activity can be evoked by mild cold stimulation, in line with its thermo-regulatory role, but also by a high-fat diet (HFD) [7–9] and insulin [10]. HFD resulted in concomitant increases in energy expenditure and BAT thermogenesis [11–14], whereas, fat loss reduced BAT thermogenesis [15–17]. The absence of BAT [18–20] or UCP1 [21,22] resulted in metabolic inefficiency leading to obesity, hyperphagia and insulin resistance [22]. Fatty acids derived from triglyceride-rich lipoproteins are the major energy carriers for brown adipocytes [23]. Activation of BAT resulted in a significant decrease of triglycerides in blood, which otherwise would be stored in the body as white fat lipids [24].

The current extensive efforts to develop drugs and methods for increasing BAT mass and activity are hindered by our current partial knowledge of the physiology of BAT *in vivo*, due in part to the absence of tools for dynamic imaging of BAT activity in real-time. FDG PET has been instrumental in advancing out knowledge of BAT *in vivo* [1–5]. FDG PET is an effective tool for detecting BAT at activation, but not at resting-state [25]. The absence of basal values hampers FDG quantitative and spatial values. Both limitations ultimately lead to a loss of important information. Therefore, a noninvasive tool is needed with better functional resolution than that available to date.

Mitochondria are the heat generators in BAT [26]. The mitochondrial membrane potential (ΔΨm) is the standard and most direct quantitative measure of the BAT heat production [27]. In the absence of heat production, the energy released by the electron transfer in the respiratory chain is used to translocate protons against the concentration gradient, thus creating a large voltage difference across the mitochondrial inner-membrane (i.e., ΔΨm). At resting-state, protons reenter the matrix via ATPase, providing the energy required for ATP synthesis. At thermogenic-state, protons bypass ATPase and reenter the matrix through UCP1 [28], and the energy stored in the concentration gradient is dissipated as heat [26, 29]. Protons reentrance through UCP1 leads to a proportional decline of ΔΨm. Thus, monitoring ΔΨm provides a direct quantitative measure of the extent of protons flux through UCP1, and thereby of the amount of heat produced by mitochondria.

The PET imaging agent ^18^F-fluorobenzyltriphenyl phosphonium (^18^F-FBnTP) is an indicator of ΔΨm [30–33]. Previous *ex vivo* studies in rats have demonstrated the capacity of ^18^F-FBnTP to detect the collapse of ΔΨm in cold-stimulated BAT [33]. A 4-hrs exposure of rats to 4°C environment, either before or after administration of ^18^F-FBnTP, resulted in a marked decrease of both uptake and retention of ^18^F-FBnTP in BAT, which was blocked by prior treatment with the ß-noradrenergic antagonist propranolol. This finding is consistent with previous observations of the tight linear relationship of ^18^F-FBnTP and ΔΨm in a spectrum of preparations from cardiomyocyte mitochondria, single cells and up to an intact-heart model [30,31]. Stepwise hyper-polarization of membrane potential in isolated mitochondria and single cells resulted in a linear increase of ^18^F-FBnTP uptake over a large range of membrane potentials, whereas selective mitochondrial depolarization, using uncoupling protocols, resulted in tight linear dose-dependent washout of ^18^F-FBnTP. Pharmacological manipulations have shown that the large majority of ^18^F-FBnTP (>80%) concentrates in the mitochondrial compartment in a ΔΨm-dependent manner, and with very low nonspecific binding (~5%). The remainder of activity was found in cytosol [30]. A similar ΔΨm-dependent fraction of ^18^F-FBnTP was measured by dynamic PET imaging of Guinea Pig’s isolated perfused intact heart [31]. Adding the uncoupler FCCP (10 μg) to the perfusion medium resulted in a linear washout of ^18^F-FBnTP—75% washout was obtained within 20 min. Uncouplers mimic UCP1 activity by translocating protons across the mitochondrial inner-membrane and into the matrix, leading to selective collapse of ΔΨm.

The present work aims at expanding our previous *ex vivo* studies, and characterizing the *in vivo* kinetics of mitochondrial uncoupling induced by controlled localized skin cooling and systemic pharmacologic stimulation, using dynamic ^18^F-FBnTP PET imaging of the rat’s BAT. At ambient temperature, ^18^F-FBnTP accumulated rapidly and extensively, generating within several minutes high-contrast images of resting BAT. Conversely, mitochondrial uncoupling induced by skin cooling or systemic administration of the ß3-noradrenergic agonist CL-316,243 resulted in an immediate washout of ^18^F-FBnTP from BAT, which was blocked by propranolol. We characterized some key variables of BAT-evoked activity, including response latency, magnitude and temporal kinetics, and demonstrated indications that rat’s BAT is a highly sensitive and rapidly responsive organ, which generates heat in an immediate, short burst of several minutes duration, while body’s core temperature remains intact.

## Materials and Methods

### Animals and materials

Brown Norway rats (6-mo old male; 250–350 g BW, n = 22) were purchased from the NIA colony. ^18^F-FBnTP was prepared in our radiochemistry laboratory with a specific activity ranging from 111 to 185 GBq/mmol (12,000–25,000 mCi/mmol), as described elsewhere [34]. Propranolol and CL-316,243 were purchased from Sigma Eldrich. Animals were acclimated at ambient temperature of 23°C for at least one week before the imaging study with *ad libitum* food and 12/12h light/dark cycle.

### PET/CT acquisition

PET data were acquired on a GE eXplore VISTA dual-ring small-animal scanner (61 slices, 0.775-mm slice thickness, 4.8-cm axial FOV, 1.1-mm FWHM). Animals were sedated throughout the PET/CT study by isoflurane (2–3%) inhalation. Sixty to ninety minutes dynamic PET scan was commenced with tail-vein administration of 37 MBq ^18^F-FBnTP (1 mCi). Images were corrected for decay, dead times, random count and scatter. Images were reconstructed using the ordered-subsets expectation maximization (OSEM) algorithm (32 subsets, 2 iterations), into a 175 x 175 x 61-pixel matrix and 0.3875 x 0.3875 x 0.775-mm voxel size. CT images were acquired immediately after the completion of the PET scan, using small animal SPECT/CT scanner (X-SPECT; Gamma Medica), which stands next to the microPET. Animals were transferred to the SPECT/CT scanner while restrained to the bed, and sedated by isoflurane inhalation. CT images were obtained at 50 kVp and 0.6 mA. Images were captured for 5 sec per view for 256 views in a 360° rotation. PET-CT image coregistration was carried out using Mirada^cd^ and Analyze^cd^ packages. In all animals, colonic temperature was monitored periodically throughout the imaging study using digital thermometer.

### Study protocols

The following PET protocols were employed:

1. ^18^ *F-FBnTP uptake and retention kinetics in BAT at room temperature (RT)*: Animals were kept warm during the scan using heating lamp (colonic temperature ≥36°C). Dynamic PET, initiated concurrently with IV administration of ^18^F-FBnTP, was carried out for up to 60 min (n = 5). Frame duration increased gradually from 10 to 180 sec.
2. *Validation of* ^*18*^ *F-FBnTP selectivity for BAT*: Dynamic PET was carried out as in protocol I (n = 3). At the completion of the PET scan, animals were quickly euthanized by isoflurane overdose, and BAT was excised surgically. Next, a 10 min static scan was carried out in same bed position, as the pre-excision scan. Time interval between scan 1 and 2 did not exceed 10 min. In a separate group of animals (n = 3), the effect of euthanasia on ^18^F-FBnTP uptake in BAT was assessed. Animals were euthanized and scanned as above, but without excision of BAT.
3. *Effect of cold stimulation on* ^*18*^ *F-FBnTP uptake kinetics in BAT*. Dynamic PET was carried for 90 min, beginning with IV administration of ^18^F-FBnTP (n = 6). First 20–30 min of the scan were acquired while the animal was kept warm using heating lamp. Next, cold stimulation was applied by turning off the heating lamp and carefully placing wrapped shredded ice on the caudal back of the animal, which extended out of the scanner gantry, for the remaining duration of the scan. Colonic temperature was kept above 28°C by carefully removing the ice bag, when necessary.
4. *Effect of propranolol on* ^*18*^ *F-FBnTP washout response to cold stimulation*: Propranolol (5 mg/kg, IP) was administered 30 min before the administration of ^18^F-FBnTP and the PET scans were acquired as in protocol III (n = 4).
5. *Effect of* the ß3-noradrenergic agonist CL-316,243 *on* ^*18*^ *F-FBnTP uptake and retention in BAT*. A 90 minutes dynamic PET was initiated concurrently with ^18^F-FBnTP IV administration (37MBq); 30 min after the start of the scan CL-316,243 (10 μg/kg) was given IV (n = 4). Both ^18^F-FBnTP and CL-316,243 were administered via a tail-vein catheter.

All animal protocols were approved by the Johns Hopkins School of Medicine’s Animal Care and Use Committee.

### Image and data analysis

Quantification of ^18^F-FBnTP uptake was carried out on coronal section of interscapular BAT (iBAT). Images were resampled to cubic voxels (0.775-mm^3^), and a medial section intersecting iBAT was selected for further analysis. Frames acquired over 10-min period just before stimulus application were summed up, representing ^18^F-FBnTP BAT basal activity, and segmented using 50% of maximum activity as cutoff value (Tmax50%). All basal iBAT voxels, visible on the segmented basal PET image and localized to low CT Hounsfield unit area, were delineated using automatic ROI. The ROI template was copied to the temporal images and mean activity was computed. Small cubic ROIs (0.775-mm^3^) were placed just outside of BAT (background activity) and on the left ventricular (LV) wall. ^18^F-FBnTP activity was expressed as [counts/mCi injected/kg BW]. Image processing was carried out by PMOD^ct^ and Analyze^ct^ packages.

### Statistical analysis

Results are expressed as mean±SD. Level of significance between different organs and conditions was calculated using 2-tailed paired t-test. P value ≤0.05 was considered to indicate statistical significance.

## Results

### ^18^F-FBnTP Strongly Accumulates in BAT at Rest

**Fig 1:**
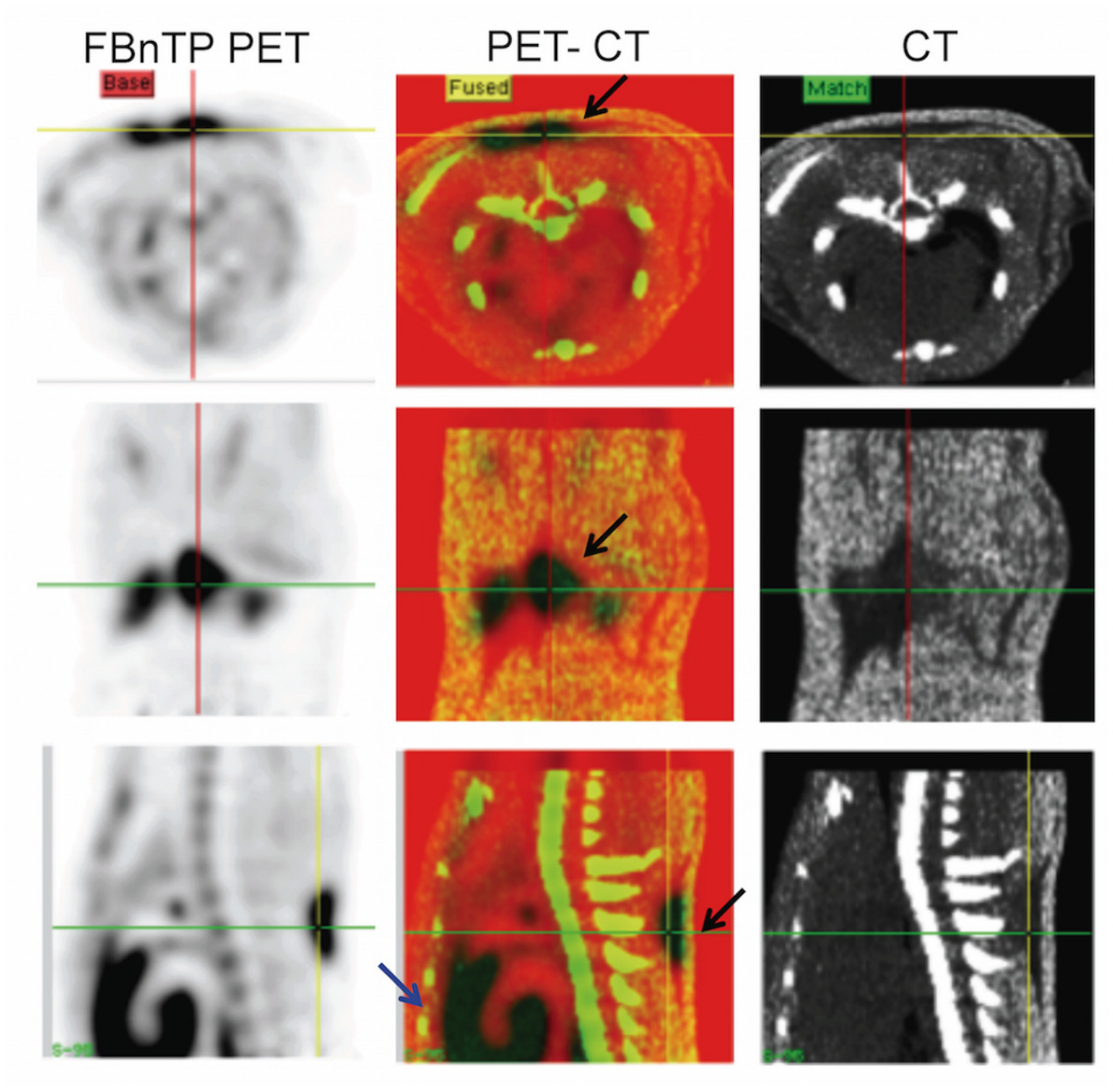
^18^F-FBnTP Uptake in Interscapular Brown Fat Depots at Room Temperature. Coregistered PET/CT images in transverse (upper panel), coronal (mid panel) and sagittal (lower panel) view, acquired in a rat at room temperature. PET images represent summed activity acquired over the 10 to 30 min time interval after ^18^F-FBnTP administration. ^18^F-FBnTP increased uptake in the interscapular area is confined to CT regions of low Hounsfield units (black arrows). Note the strong uptake of ^18^F-FBnTP in BAT, similar to that seen in heart (blue arrow).

**Fig 2:**
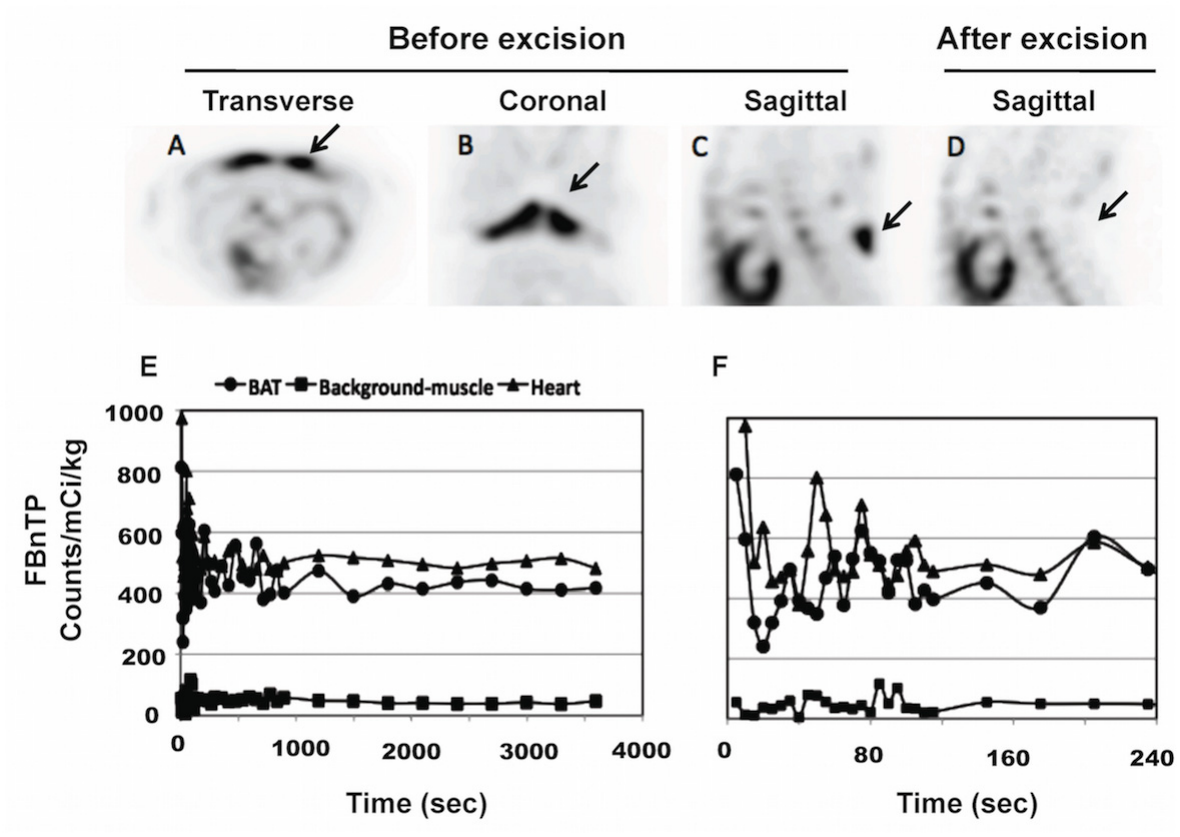
^18^F-FBnTP Uptake Kinetics and Selectivity to BAT at Rest. ^18^F-FBnTP PET images acquired in same animal before (A—C) and after surgical excision of BAT (D), and related time-activity curves (E—F). Note the lack of uptake in the interscapular area after BAT excision (D). Images in (A to C) and (D) represent PET scans acquired in the same animal at an interval of 12 min. Chart in (E) represents ^18^F-FBnTP time activity curve generated from the same animal in (A to C). Chart in (F) is zooming of the first 240 sec in (E). Y-axis in (F) has same unit value and scale as in (E). Note the strong ^18^F-FBnTP uptake in BAT, which is similar to that in the heart, and 8 times greater than background activity (E). ^18^F-FBnTP reaches plateau concentration in BAT within less than a minute (F).

Dynamic PET was carried out in BN rats (n = 5), as outlined in Protocol I. In all animals, a strong preferential accumulation of ^18^F-FBnTP was found in the interscapular area, localized to regions of low Hounsfield units as identified by coregistered CT (Fig 1). To validate that ^18^F-FBnTP uptake is restricted to iBAT, PET scan was acquired in same-bed position before and after excision of BAT (Protocol II, n = 3). ^18^F-FBnTP uptake in iBAT was observed before, but not after excision (Fig 2). Total time interval between the pre- and post-excision scans was less than 8 min. In a separate group of animals, we examined the effect of euthanasia on ^18^F-FBnTP retention in BAT (n = 3). Animals underwent same procedure as above, but without excision of BAT. In all animals, BAT was clearly visible on PET images, albeit ^18^F-FBnTP uptake was slightly lower (8.1%±7.5%; P<0.281), compared to that measured in the living animal.

Analysis of time activity profiles revealed a very rapid and extensive accumulation of ^18^F-FBnTP in BAT.^18^F-FBnTP peak activity in resting BAT was obtained within 10 to 20 seconds and plateaued within a few minutes (Fig 2F).^18^F-FBnTP maintained prolonged steady-state concentration in BAT for the entire scan duration (Fig 2E). ^18^F-FBnTP plateau activity in BAT at ambient temperature ranged between 471 and 635 (522.2±108.3; n = 5). ^18^F-FBnTP BAT- to-background ratio was 6:1 to 10:1. ^18^F-FBnTP plateau concentration in BAT was similar to that of the heart (Fig 2E). Mean BAT-to-heart ratio was 0.88±0.13 (n = 5). In large and small animals, heart is a major target organ of ^18^F-FBnTP, second only to kidney [32, 35].

### Cold Stimulation Results in an Immediate—Washout of ^18^F-FBnTP from BAT

The effect of localized skin cooling on ^18^F-FBnTP retention in BAT was studied using 90-min dynamic PET scan (Protocol III, n = 6). ^18^F-FBnTP was administered IV, and the first 20 or 30 min of the dynamic scan were acquired while the animal was kept warm, using heating lamp. Cold stimulation was applied for the remaining scan time. Colonic temperature was monitored throughout scan time.

**Fig 3:**
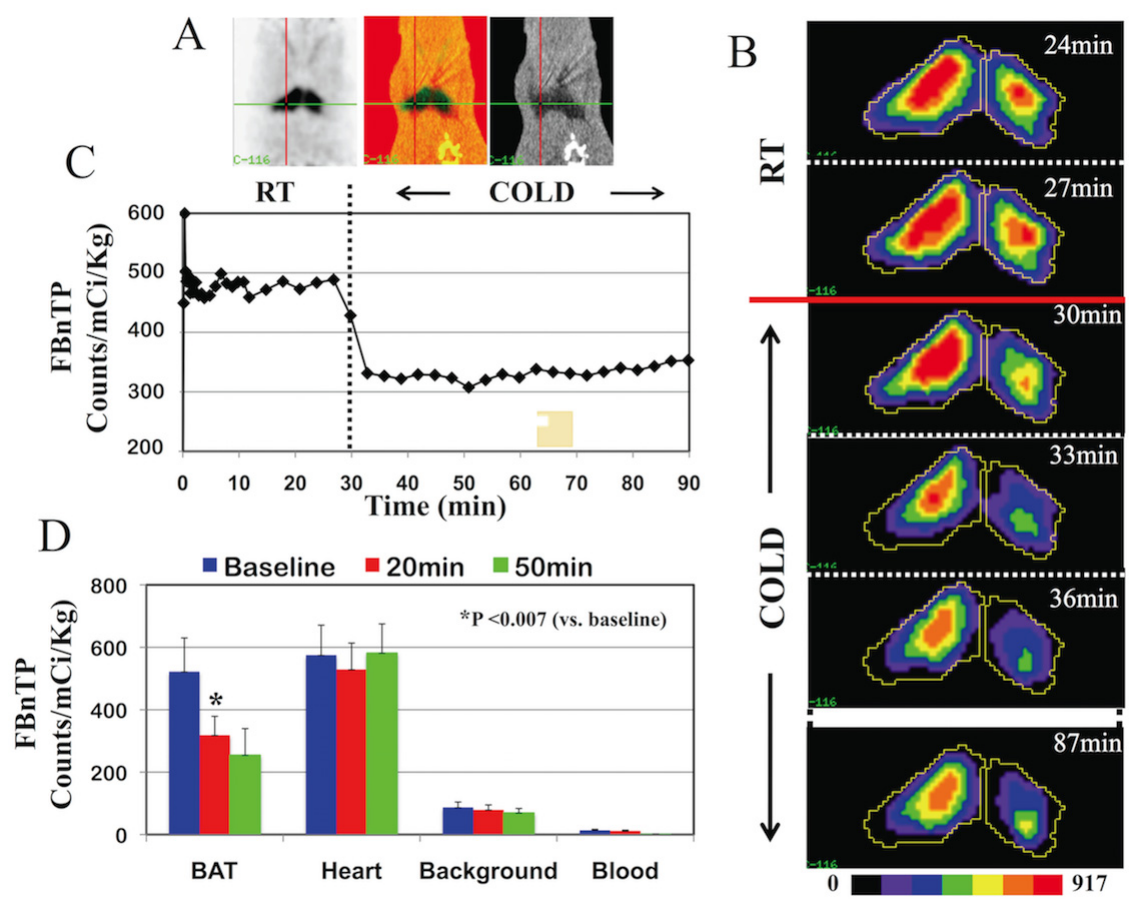
Cold-induced Mitochondrial Uncoupling Elicits an Immediate ^18^F-FBnTP Washout from BAT. (A) Coronal PET/CT images of BAT acquired at room temperature. (B) ^18^F-FBnTP PET images of BAT before and during cold stimulation. Images are segmented using max_50%_ cutoff value. Each image represents summed activity over 3 min. Beginning of acquisition time of each frame is indicated in upper right corner. Cold stimulation started at the 30 min point of the scan. (C) ^18^F-FBnTP time activity curve generated from same animal depicted in (B). (D) ^18^F-FBnTP mean uptake measured on 10-min image frame, acquired just before (baseline) and 20 and 50 min after the start of cold stimulation (mean±SD, n = 6). Note the immediate sharp decrease of ^18^F-FBnTP uptake upon application of cold stimulation (B and C), and small, but insignificant washout at later time points (D), as well as lack of effect on uptake in heart (D).

Contrary to the prolonged steady-state retention observed at room temperature (RT), cold stimulation resulted in a rapid washout of ^18^F-FBnTP from BAT. Fig 3 depicts an example of ^18^F-FBnTP PET images and related time-activity curve acquired before (RT) and during cold stimulation (COLD). Each image represents summed activity over 3 min, and start-times of each frame is indicated at the upper right corner (Fig 3B). ^18^F-FBnTP washout kinetics can be evaluated qualitatively by the PET images (Fig 3B), and quantitatively by the time activity curve (Fig 3C).

Time-to-onset of washout was in the few-minutes range (2.2±1.3 min, n = 6). The duration of the early washout phase ranged from 2 to 8 min (2.71±2.42 min). Extent of cold-induced washout of ^18^F-FBnTP from BAT, expressed as percentage of mean basal activity, was 39.1%±14.4% (n = 6, P <0.007) (Fig 3D). The kinetics of the late response phase varied between animals. Both, slow or no washout, were observed in different animals. An additive but insignificant washout of 11.1%±17.5%, (n = 6; P <0.18) was measured at 50 to 60 min, compared to 20 to 30 min post-administration time interval (Fig 3D).

FBnTP retention in the myocardium was not significantly affected by skin cooling (Fig 3D). A slight washout of ^18^F-FBnTP was observed in 2 out of 6 rats. Unlike the abrupt washout observed in BAT, the clearance from heart was linear, if at all.

### The Bulk of ^18^F-FBnTP Washout Occurs while Body Core Temperature Remains Intact

**Fig 4:**
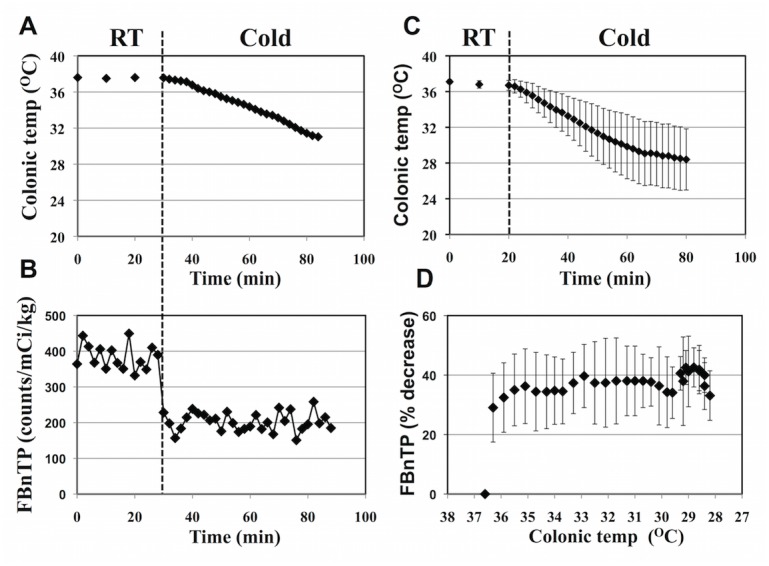
^18^F-FBnTP Washout Response Precedes Change in Body Core Temperature. Colonic temperature (A) and ^18^F-FBnTP uptake kinetics (B) monitored in same animal before and during skin cooling. (C) Mean change in colonic temperature induced by cold stimulation (mean±SD, n = 6). (D) Extent of ^18^F-FBnTP washout response correlated with colonic temperature (n = 6). Note the sharp washout of ^18^F-FBnTP, which nearly completed before significant change in colonic temperature was attained (D).

Onset of ^18^F-FBnTP washout from stimulated BAT occurred before significant change in colonic temperature was observed (Fig 4A and 4B). The cold stimulation protocol employed in the present study induced a typical linear decrease of colonic temperature at rate of 0.17±0.05°C/min (n = 6) (Fig 4C). In all animals, the most of the early steep washout phase was maintained while colonic temperature was ≥35.8°C (Fig 4D). Decreasing of colonic temperature below 35.8°C and into the shivering range did not elicit an additional washout of ^18^F-FBnTP (Fig 4D).

### ^18^F-FBnTP Washout Response Is Mediated by the Noradrenergic System

**Fig 5:**
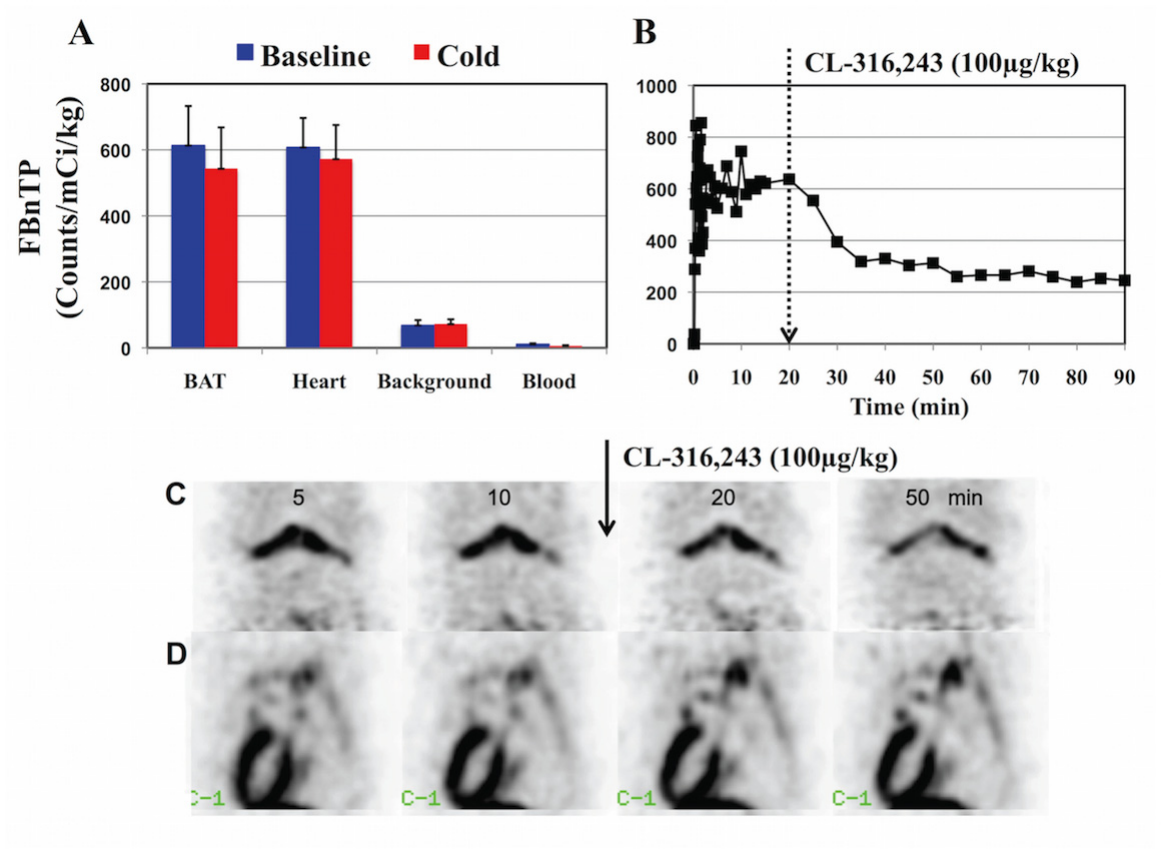
Noradrenergic Mediation of ^18^F-FBnTP Washout Response. (A) Effect of propranolol ^18^F-FBnTP-washout response to cold stimulation. Propranolol blocked cold-induced ^18^F-FBnTP washout from BAT. A slight but insignificant decrease (9.6%±11.6%; P<0.21) was measured. (B) Effect of the ß3-AR agonist CL-316,243 on ^18^F-FBnTP retention in BAT. Administration of CL-316,243 (100 μg, IV) resulted in ^18^F-FBnTP washout from BAT with kinetics similar to those observed during cold stimulation. (C) ^18^F-FBnTP PET imaged of BAT before and after administration of CL-316,243. Both PET images in (C) and time activity curve in (B) were taken form same rat’s BAT. Note the CL-316,243-induced washout kinetics, which are similar to those observed during localized skin cooling. (D) CL-316,243 hardly affected ^18^F-FBnTP retention in heart muscle.

Two sets of studies were carried out to examine the role of the ß-noradrenergic receptor system in ^18^F-FBnTP washout response. First, the effect of the sub-type non-selective ß-noradrenergic antagonist propranolol was studied (Protocol IV, n = 4). Propranolol (5 mg/kg, IP) was administered 30 min before commencement of ^18^F-FBnTP PET dynamic scan. First 20 min of the scan were acquired at room temperature, and cold stimulation was employed for the remaining scan time. Administration of propranolol has had two effects. (i) ^18^F-FBnTP basal uptake was 17.6% greater in propranolol-treated, compared to non-treated rats (Prop 613.5±121.3; no-Prop 522.2±108.3, P <0.052); (ii) Propranolol significantly reduced ^18^F-FBnTP washout response to cold. ^18^F-FBnTP washout from BAT, measured as the mean decrease over the time interval of 50–60 min of cold stimulation, was significantly lower in propranolol-treated, compared to non-treated rats (9.6%±11.6% (P<0.21) vs. 39.1±14.4% (P<0.007), respectively) (Fig 5). ^18^F-FBnTP retention in heart was not affected by propranolol treatment.

Second, the effect of the ß3-noradrenergic selective agonist CL-316,243 on ^18^F-FBnTP uptake in BAT was documented using 90 min dynamic PET (Protocol V, n = 3). CL-316,243 (100 μg/kg) was administered IV via tail vein 30 min after the commencement of the 90min dynamic ^18^F-FBnTP PET. Administration of CL-316,243 resulted in immediate ^18^F-FBnTP washout from BAT, compared to baseline activity (Fig 5). Washout response kinetics were similar to those observed during cold stimulation. CL-316,243 had no effect on ^18^F-FBnTP retention in heart (Fig 5).

## Discussion

Mitochondrial respiration and ΔΨm are the two most established interrelated measures of thermogenesis' *in vitro*. Heat is produced by the protons flux down the concentration gradient, resulting in loss of ΔΨm and disengagement of substrate phosphorylation and ATP synthesis. This aberrant condition imposes a hypoxia-like condition and a compensatory increase of the organelle’s oxygen utilization. Perfusion studies using O-15 PET documented the increase of oxygen utilization in BAT during thermogenesis, but not the kinetics of the BAT response [36]. In the present study, we demonstrated the advantage of targeting ΔΨm using ^18^F-FBnTP PET, for dynamic imaging of BAT thermogenesis. ^18^F-FBnTP PET provided evidence that BAT is a highly responsive organ in the living animal, and that the bulk of heat (i.e., mitochondrial uncoupling) is generated as a short burst, of few-to-several minutes, immediately upon stimulation.

Three key requirements are essential for an imaging compound to act as a reliable noninvasive indicator of ΔΨm and thermogenesis. (i) Linear dose-dependent relationship with ΔΨm over a wide range of membrane potentials. This characteristic determines the quality of resting BAT image; the benchmark for measuring alterations of ΔΨm during thermogenesis. Importantly, mitochondrial capacity to produce heat is dictated by the extent of proton gradient (i.e., ΔΨm). The greater the protons gradient, the greater ΔΨm and the potential capacity for heat production. Some potentiometric probes plateau at high ΔΨm values, and therefore may not provide a true measure of the tissue's capacity for thermogenesis [26]. (ii) The fraction of the probe molecules concentrating in the mitochondrial compartment should be in a labile form, and readily expelled upon ΔΨm decline. This characteristic is crucial for a reliable monitoring of rapid changes in ΔΨm, as these occur in BAT during activation. *In vitro*, mitochondrial uncoupling is a rapid event in the seconds-range. (iii) It has to maintain low nonspecific binding. Once UCP1 are opened, proton reentrance to matrix is expected to be maintained until concentration gradient is completely abolished, leading to collapse of ΔΨm to near zero values and complete expulsion of the potentiometric probe. This highlights the need for minimal nonspecific binding. Most potentiometric probes are lipophilic, and nonspecific binding to membrane constituents may mask the decline of ΔΨm during thermogenesis. [33].

The results of the present *in vivo* study, together with previously-obtained *in vitro* and *ex vivo* data, suggest that ^18^F-FBnTP complies with the above requirements. First, at rest, when ΔΨm is intact, ^18^F-FBnTP accumulated extensively in BAT. ^18^F-FBnTP BAT-to-background contrast was > 6:1. Whole-body PET scans in large and small animals have shown that ^18^F-FBnTP is targeting body organs, in proportion to their mitochondrial content, and heart uptake is second only to that of the kidney [30,32]. In the present study, ^18^F-FBnTP uptake in BAT was similar to that in heart [30,32]. ^18^F-FBnTP avidity for mitochondria is because ΔΨm is much greater than the plasma membrane potential (200–240 mV vs. 30–60 mV, respectively [37]). According to Nernst Equation, each 60 mV difference results in 10-fold increase of the potentiometric probe uptake. In carcinoma cells, ^18^F-FBnTP concentration in the mitochondrial compartment was approximately 10^4^ times that in the cytosol and comprised >80% of total cellular uptake [30]. Under the assumptions of 150 mV for ΔΨm and a matrix volume of 1% of total cytoplasm, 75% of Nernstian probe is expected to concentrate in the mitochondria in a ΔΨm-dependent manner [38], similar to that observed for ^18^F-FBnTP in carcinoma cells [30].

Second, mitochondrial depolarization induced by localized skin cooling and systemic activation of ß3-noradrenergic receptors, resulted in an immediate, abrupt washout of ^18^F-FBnTP from BAT. The short latency and rapid washout rate indicate that the fraction of ^18^F-FBnTP concentrated in mitochondria is labile, and readily expelled upon decline of ΔΨm. Third, *in vitro* studies of pharmacologic manipulations of cytoplasma and mitochondrial membrane potentials have shown that ^18^F-FBnTP maintains very low nonspecific binding (~5%) [30].

The receptor mechanism underlying ^18^F-FBnTP evoked response was validated using activation (CL-316243), and suppression (propranolol) of the β-adrenergic receptor (AR) system. The results of both studies supported β-adrenergic mediation of ^18^F-FBnTP washout response. The β3-AR-specific agonist CL-316243 elicited ^18^F-FBnTP washout from BAT similar to the kinetics observed upon cold stimulation, including short onset time and rapid washout rate. In rodents, the β3-AR is found nearly exclusively on brown adipocytes, and treatment with CL-316243 substantially increases energy expenditure [39–40]. The administration of the adrenergic antagonist propranolol strongly mitigated ^18^F-FBnTP washout from stimulated BAT. This further bolsters the linkage of ^18^F-FBnTP response to cold-induced mitochondrial uncoupling and heat production.

### BAT Response Kinetics

^18^F-FBnTP PET has shed light on some key aspects of the physiology of BAT evoked activity. First, ^18^F-FBnTP PET has shown that BAT is a rapidly responsive organ. Both, cold- and ß3-AR stimulation caused a nearly immediate washout of ^18^F-FBnTP with a response onset time in the few-minute range. This finding is consistent with *in vitro* observations in isolated mitochondria and brown adipocytes. Administration of noradrenalin to the incubation medium resulted in an immediate mitochondrial uncoupling, expressed by sharp increase of mitochondrial respiration [26]. Maximum respiration was obtained within 2 min [26]. Similar results were obtained in brown adipocytes [41]. The present study suggests that the rapid kinetics of mitochondrial uncoupling observed *in vitro* are preserved in the intact animal. The results of the present study are also consistent with whole-body measurement of respiration in rats. Cold [42] and noradrenergic agonists [43] resulted in an early abrupt increase of whole-body oxygen utilization in the few-minutes range.

The present study provides indications, in the intact animal model, that BAT is only partially activated by cold stimulation. The skin cooling protocol employed in the present study resulted in an abrupt drop of approximately one-third of ^18^F-FBnTP, compared to basal uptake. Partial clearance of ^18^F-FBnTP from BAT was obtained by systemic activation of ß3-AR. Magnitude of ^18^F-FBnTP washout may serve as an index of both extent of decline of DY and amount of mitochondria recruited for heat production. However, a rigorous assessment of extent of activation should take into account the distribution kinetics of ^18^F-FBnTP once released from uncoupled mitochondria. In the present study, both cold and pharmacological activation of ß3-AR resulted in bimodal washout; an abrupt decline of activity over a short time (2–8 min), which was followed by steady state concentration for the remaining scan time (60–70 min). Dynamic PET of isolated perfused heart has shown that mitochondrial uncoupling induced by 10 μM FCCP resulted in linear, rather than bimodal, washout of ^18^F-FBnTP from the LV wall, and the extent of washout was significantly greater than that observed in stimulated BAT—50% to 75% depletion of uptake was obtained within 20 min. This suggests that ^18^F-FBnTP washout kinetics observed in the present study are organ specific, and may point to additional players, such as^18^F-FBnTP re-distribution from uncoupled (i.e., thermogenically active) to yet coupled (i.e., inactive) mitochondria. Thus, the slow, late washout may represent the net result of two opposing dynamics, re-uptake of ^18^F-FBnTP to mitochondria of yet intact ΔΨm, which may mask diffusion of the imaging agent from tissue to the blood pool. Furthermore, carful quantitative assessment of magnitude of BAT activation requires examining the contribution of the increased blood flow to ^18^F-FBnTP washout kinetics. Efforts to identify the forces involved in ^18^F-FBnTP early- and late-phase dynamics using dose- and duration-dependent protocols of pharmacologic and cold stimulation, respectively, are in progress.

The common school of thought holds that BAT thermo-homeostatic role is in the non-shivering temperature range. Accordingly, we explored the effect of shivering colonic temperature on mitochondria uncoupling in BAT. Our results suggest that mitochondrial depolarization in BAT is indeed confined to non-shivering conditions. Drop of body core temperature to the shivering range has marginal effect, if at all, on BAT mitochondria. This provides an important physiological validation of the non-shivering adaptive role of BAT in the living animal.

## Conclusion

Monitoring ΔΨm using ^18^F-FBnTP PET provided important insights into key aspects of BAT thermogenesis *in vivo*. ^18^F-FBnTP PET depicts rodent’s BAT as a highly sensitive and rapidly responsive organ, emitting the bulk of heat in a short-lasting burst, over the first minutes of the cold stimulation. The present study also provide physiological evidence in support the the non-shivering adapative role of BAT. Prolonogation and decrease of core tempearture to shivering range has mariginal additive effect on short-term mitochondrial recrutiment. The capacity of ^18^F-FBnTP PET to monitor BAT response kinetics in real-time, allowed us to identify and quantify principal variables of thermogenesis, including response onset time, magnitude and kinetics. As such, ^18^F-FBnTP PET provides a powerful research platform for the study of BAT physiology *in vivo*, as well as a novel set of quantitative metrics, which can be helpful for identifying therapeutic targets at the mitochondrial level, for developing of means to maximize BAT mass and activity, thus enabling sensitive and accurate assessment of their efficacy.
